# Analysis of Human Information Recognition Model in Sports Based on Radial Basis Fuzzy Neural Network

**DOI:** 10.1155/2022/5625006

**Published:** 2022-05-26

**Authors:** Tong Li, Longfei Ren, Fangfang Yang, Zijun Dang

**Affiliations:** ^1^Yong In University, Yongin 17092, Republic of Korea; ^2^Honam University, Gwangju 62399, Republic of Korea

## Abstract

In sports, because the movement of the human body is composed of the movements of the human limbs, and the complex and changeable movements of the human limbs lead to various and complicated movement modes of the entire human body, it is not easy to accurately track the human body movement. The recognition of human characteristic behavior belongs to a higher level computer vision topic, which is used to understand and describe the characteristic behavior of people, and there are also many research difficulties. Because the radial basis fuzzy neural network has the characteristics of parallel processing, nonlinearity, fault tolerance, self-adaptation, and self-learning, it has the advantage of high recognition efficiency when it is applied to the recognition of intersecting features and incomplete features. Therefore, this paper applies it to the analysis of the human body information recognition model in sports. The research results show that the human body information recognition model proposed in this paper has a high recognition accuracy and can detect the movement state of people in sports in real time and accurately.

## 1. Introduction

Among the many sports application fields based on artificial intelligence technology, sports video analysis is one of the more popular branches. The main reason is that sports videos have a wider audience and huge market potential. In addition to traditional TV users and sports enthusiasts, sports programs are also popular among emerging media users such as mobile terminals and webcasting and are favored by some sports professionals (coaches, athletes, referees, and scouts) [1–9]. Sports video can not only provide viewers with a richer game experience but also provide technical and tactical analysis for sports professionals. With the rapid development of mobile devices and the Internet, people's demand for sports videos has also shifted from simple viewing to diversified needs such as game statistics, highlight video clips, specific event detection, video content enhancement, technical and tactical analysis, and auxiliary training, auxiliary judgment. From the perspective of academic research, realizing the above-mentioned diversified user needs requires the comprehensive use of many technologies in various fields such as visual technology, multimedia, pattern recognition, and machine learning, and research in the field of sports video analysis will also promote these technologies. *Further development*. The academic exchanges on sports video analysis are also very active. The latest research results on sports video analysis are published every year in various academic journals and conferences related to multimedia, artificial intelligence, computer graphics, etc. [10–15].

In the field of feature recognition technology research, a variety of feature recognition methods have appeared successively. All these methods first give the pattern definition of the feature and then identify it by pattern matching. However, due to the complexity of pattern definition of intersecting features and incomplete features, the above methods have lower recognition efficiency for intersecting features and incomplete features.

In recent years, in the field of computer vision, more and more researchers have shown great interest in human motion recognition. The movement of the human body is dynamic, so the method for recognizing human action is also based on the dynamic method. So far, there are mainly three kinds of mature and reliable methods: the method based on template matching, the method based on grammar, and the method based on the probabilistic graphical model.

### 1.1. Template Matching Method

The method based on template matching is to convert the image sequence into one or a group of templates and then identify the behavior to be identified by matching the known template. The images are first transformed into motion energy image (MEI) and motion history image (MHI), and then, Mahalanobis distance is adopted as a measure of template similarity. Although this method requires a small amount of computation, it is sensitive to changes in time intervals and is less robust.

### 1.2. Grammar-Based Approach

Human behavior has a strong hierarchical and combinatorial structure, which is similar to the grammatical organization structure, so people begin to study methods based on grammatical models to describe human behavior. The semantics of human behavior is a high-level meaning based on behavior characteristics, and it is a highly abstract description form to describe behavior attributes. This method can accurately describe the true meaning of behavioral objects and easily transition to natural language.

### 1.3. Methods Based on Probabilistic Graphical Models

Probabilistic graphical models are divided into two categories: directed graphical models and undirected graphical models. Typical directed graphical models are hidden Markov models, and typical undirected graphical models are mainly conditional random fields.

Artificial neural network, also known as neural network, is a mathematical model for information processing and calculation by imitating the characteristics of biological neural network behavior [16–18]. It works through the connection between artificial neurons. In many cases, it can use external information to change its own structure, and it has strong autonomous learning and adaptive capabilities. Neural networks often use modeling methods to describe the complex relationship between input and output or to explore the pattern of research data. It is one of the commonly used tools for statistical nonlinear data. The neural network has a strong self-learning ability. For example, when performing image recognition and classification, we only need to input the processed image samples into the trained neural network, and the neural network will use its own learning function to perform image recognition and classification. In addition, the self-learning function of the neural network also plays a very important role in forecasting. It can effectively forecast the market economy and enterprise benefits and has a wide range of applications. In addition, the neural network also has a feedback function. In real life, when an optimal solution to a complex problem is processed, a large amount of calculation is required. At this time, the feedback function of the neural network can be used to calculate some objects and find the optimal solution to the problem [19–23].

Because the neural network has the characteristics of parallel processing, nonlinearity, fault tolerance, self-adaptation, and self-learning, it has the advantage of high recognition efficiency when it is applied to the recognition of intersecting features and incomplete features [24–26]. Therefore, neural network technology is introduced to the field of 3D feature recognition, and the established neuron is a classifier for linearly separable patterns. Through teaching training, basic features such as grooves, through grooves, and through holes as well as incomplete features can be recognized, but complex features such as intersecting features such as cross grooves cannot be recognized. To end this issue, a multilayer perceptron neural network is applied to identify features, which can identify more complex features such as holes through edges and through points.

RBF neural network is widely used in many places, and it is nonlinear multilayer feedforward networks, both are approximators, which can approximate any continuous, nonlinear function. Similarly, for any RBF neural network, there will always be a BP neural network corresponding to it, but there are many differences between the two [27–29]. Therefore, in this paper, first of all, the detection and tracking of moving objects: the detection of moving objects is realized through background modeling. On this basis, the tracking of moving objects is realized so as to realize the extraction of the spatiotemporal region where the moving objects are located, which is used as the object of subsequent human behavior recognition; then, the image feature extraction: the main research visual features include multiscale and multidirectional Gabble feature group, shape context feature, visual keyword histogram, so as to realize the visual description of human action features.

## 2. Radial Basis Fuzzy Neural Network

Each neuron on the hidden layer represents a radial basis function, which is usually a Gaussian function. This function is mainly determined by two parameters: the field center and the field width.

The radial basis function is a real-valued function whose value depends only on the distance from the sample to a point *w* in the sample space, that is,

(1) $$\begin{array}{c} g\left(x,w\right)=g\left(\left\|x-w\right\|\right), \end{array}$$

where *x* is the input of the sample and *w* is the center of the kernel function. Therefore, any function *g* that satisfies the following properties is called a radial basis function.

(2) $$\begin{array}{c} g\left(x\right)=g\left(\left\|x\right\|\right). \end{array}$$

The standard radial basis function generally uses the Euclidean distance also known as the Euclidean radial basis function. The radial basis function used in this paper is a Gaussian kernel function built on the basis of the normal distribution, and the expression is as follows:

(3) $$\begin{array}{c} g\left(x,w,\sigma \right)=\exp\left(\frac{-{\left\|x-w\right\|}^{2}}{2\sigma^{2}}\right). \end{array}$$

**Figure 1:**
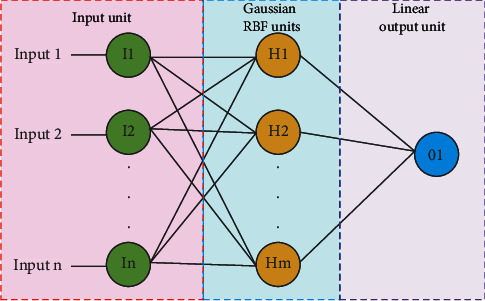
The structure of the RBF neural network.

Among them, *σ* is the width parameter of the Gaussian function, which controls the radial range of the function. The structure of the RBF neural network is shown in Figure 1.

According to the structure of the radial basis neural network, it can be seen that it has the following two characteristics for the fitting model: First, it can well solve the nonlinear relationship between sample features and sample labels. Second, it can effectively regress the correspondence between training sample features and their labels. Therefore, this paper chooses to use radial basis neural network regression to fit the human motor unit strength estimation model.

In addition, this paper sets the mean square error as the cost function in the training process. The error is quantified in the regression process by calculating the mean squared error between the true and predicted values, and the model parameters are optimized based on this error. To ensure the validity of the model, the calculation formula is

(4) $$\begin{array}{c} MSE=\frac{1}{n}\sum_{i=0}^{n}{\left(y_{i}-{\hat{y}}_{i}\right)}^{2}, \end{array}$$

where *yi* is the motion unit intensity label corresponding to the *i*th sample.

In this paper, when training the RBF neural network, K-means is used, that is, the learning method of the self-organized selection center. This learning method can be divided into two processes. The first process is the unsupervised learning process, which is used to calculate the hidden layer base center and variance of the function, and this is the process of autonomous learning of the neural network; the second process is the learning stage with a tutor, the purpose is to find the weights from the hidden layer to the output layer.

In the learning of the RBF neural network, the Gaussian function is often used as the radial basis function of the hidden unit. In this case, the activation function of the RBF neural network can be expressed as follows:

(5) $$\begin{array}{c} R\left(x_{p}-c_{i}\right)=\exp\left(-\frac{1}{2\sigma^{2}}{\left\|x_{p}-c_{i}\right\|}^{2}\right). \end{array}$$

The output of the RBF neural network is thus

(6) $$\begin{array}{c} y_{j}=\sum_{i=1}^{h}w_{ij}\exp\left(-\frac{1}{2\sigma^{2}}{\left\|x_{p}-c_{i}\right\|}^{2}\right). \end{array}$$

Among them, *ω* is the weight from the hidden layer to the output layer, and *x*_*p*_ is the sample of the *p*th input.

(7) $$\begin{array}{c} x_{p}={\left(x_{1}^{p},x_{2}^{p},\ldots ,x_{n}^{p}\right)}^{T}. \end{array}$$

Let *d* denote the expected sample output, then the variance of the basis function is

(8) $$\begin{array}{c} \sigma =\frac{1}{p}\sum_{j}^{m}{\left\|d_{j}-y_{j}c_{i}\right\|}^{2}. \end{array}$$

The learning algorithm steps are as follows:

**Step 1 .**

Find the center *C* of the basis function based on the *K*-means clustering method.

(1) Initialize the network: randomly select *h* training samples as *K*-means clustering centers:
  (9) $$\begin{array}{c} c_{i}\left(i=1,2,\ldots ,h\right). \end{array}$$
(2) Group the input samples according to the nearest neighbor principle: according to the Euclidean distance between *x* and the cluster center *c*, group *x* into the cluster set of training samples.
  (10) $$\begin{array}{c} \vartheta_{p}\left(p=1,2,\ldots ,P\right). \end{array}$$
(3) Readjust the cluster center: count the average value of the training samples in all cluster sets to obtain a new cluster center *c.* If the cluster center *c* remains unchanged, the obtained *c* is the RBF neural network basis function center, otherwise return to (2) for the next round of basis function center calculation.

**Step 2 .**

Calculate the variance. Here, we choose the Gaussian function as the basis function of the RBF neural network, and the variance is solved:

(11) $$\begin{array}{c} \sigma_{i}=\frac{c_{\max}}{\sqrt{2h}}, \end{array}$$

where *c*_max_ represents the maximum length between all selected cluster centers.

**Step 3 .**

Use the least squares method to calculate the weights from the neurons in the hidden layer to the neurons in the output layer. The formula is as follows:

(12) $$\begin{array}{c} \omega =\exp\left(\frac{h}{c_{\max}^{2}}{\left\|x_{p}-c_{i}\right\|}^{2}\right). \end{array}$$

In fact, when learning the RBF neural network, the three parameters of the hidden node basis function center, the variance of the Gaussian function, and the weight from the output layer to the hidden layer play a very important role in the performance of the network. Due to the limited personal ability, this paper mainly studies the role of the weights from the hidden layer to the output layer in the neural network. The RBF neural network generally obtains the weights from the hidden layer to the output layer through iterative training, but the more the iterations, the longer the network training time and the more unstable the network performance. Therefore, this paper uses the genetic algorithm to optimize the weights of the RBF neural network, and the purpose is to make the function have better nonlinear function approximation ability, ensure the stability of the network, and improve the correct recognition rate of human actions.

## 3. Human Body Information Recognition

**Figure 2:**
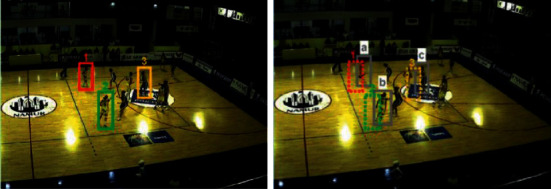
Basketball player.

This part takes basketball as an example by using the RBF neural network. Given two consecutive frames and the coordinate positions of players in each frame, the core problem of multiplayer tracking is how to effectively solve the problem of player matching between frames. As shown in Figure 2, suppose three players are tracked in frame *t*−1 (red, green, and yellow rectangles represent players 1, 2, and 3, respectively), and three new players are tracked in frame *t*. The detected target player (represented by the solid grey rectangle). For ease of description, the three identified targets in frame *t*−1 are labeled as 1, 2, and 3, and the three unknown players in frame *t* are labeled as *a*, *b*, and *c*. The core problem of the multiplayer tracking problem is to match *a*, *b*, *c* with 1, 2, and 3 one by one so that the identity of the same player is always consistent during the tracking process. In this regard, this chapter intends to obtain more accurate correlation matching results based on the extraction of players' appearance depth features and motion features so as to alleviate the phenomenon of player identity exchange during the tracking process. The previous methods mainly perform correlation matching by directly calculating the similarity of the appearance features of the two targets in the matching pair. However, in sports videos, players of the same team often wear the same color jerseys, so their appearance characteristics are very similar, making matching based on player appearance characteristics ineffective. In recent years, researchers have observed that, compared to individual features, the contextual information of objects helps to further improve feature representation. In this regard, this chapter proposes a multitarget tracking method based on pose alignment features and neural networks, which can effectively alleviate the problem of easy exchange of tracking identities caused by similar appearances. First, construct a context graph for each player and its surrounding players (node attributes are pose alignment features, and edges are connections between adjacent frames) and then use the powerful modeling ability of graph convolutional neural networks for relational features to connect adjacent such that the contextual information of the player is integrated into a new feature of the target player, which is referred to as the contextual feature here. Compared with the traditional individual features, this contextual feature that integrates the surrounding neighbor information is significantly stronger in feature representation.

**Figure 3:**
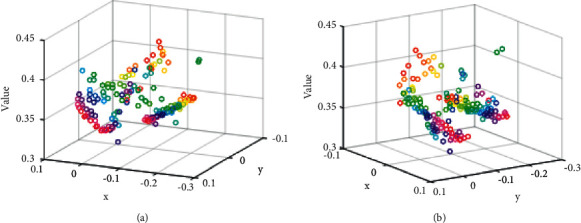
Different values versus *x* and *y*. (a) Value variation with low data and (b) value variation with large data.

For each player image block, first extract its global feature map and attitude heat map. The former is extracted by the ResNet50 network trained on the ImageNet data set, and the latter is extracted by the attitude estimation model. Among them, *C*, *H*, and *W* represent the channel number, height, and width of the feature map, respectively; *K* is the number of human joint points; and *i* is the *i*th joint point. Subsequently, the appearance features of the target player will be divided into two branches: the global feature branch and the pose-aligned feature branch. In the global feature branch, the global feature map can be transformed into a global feature vector after a simple global average optimization layer. For the pose alignment feature branch, a set of subfeature vectors corresponding to the joint points of the human body can be obtained by performing a dot product operation on the pose heat map and the global feature map and then also through the average pooling operation. In addition, the *i*th joint points that cannot be detected will be set to all 0s accordingly. It can be seen that *m* here is equivalent to a weighting coefficient and can guide the transformation of global features into joint point region features. Next, the above-mentioned subfeature vectors are further subjected to global maximization pooling, and the so-called pose-aligned feature vectors are obtained. This pose-aligned feature representation method has been proven effective in tasks such as person reidentification.  Figure 3 shows different values versus *x* and *y*.

**Figure 4:**
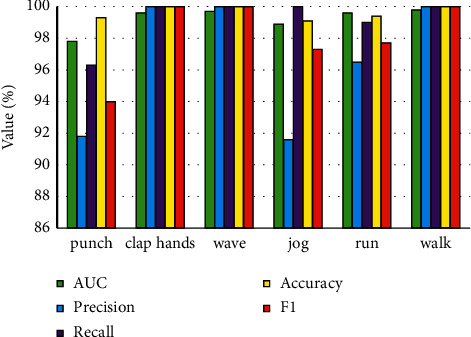
Right action recognized.

**Figure 5:**
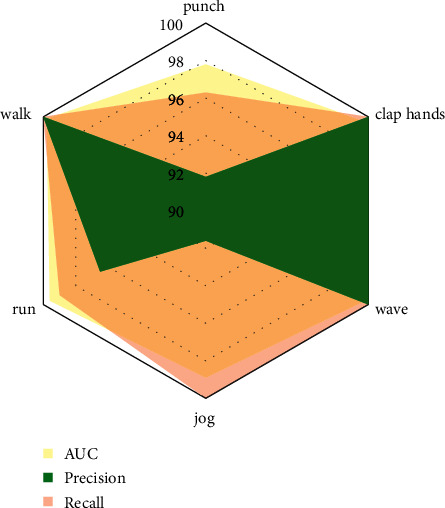
Comparison of each method.

The core of the detection-based multitarget tracking method is to solve the problem of association matching between the detection of the current frame and the existing track. To solve this problem, it is necessary to obtain the appearance features (individual features, context features, etc.) or motion features (coordinate position, speed, etc.) of each target and use this as a basis to calculate the similarity between the objects to be matched. From the above content of this chapter, there are mainly four types of information that can be used for association matching: (1) similarity information based on contextual features, (2) similarity information based on individual player characteristics, (3) Mahalanobis distance based on motion state, and (4) the intersection over union (IoU) between the trajectory-based Kalman prediction box and the player detection box in the current frame. How to make full use of the above information to obtain a reliable matching result is not easy. This is because the above information is not absolutely reliable, especially in some complex situations, such as short-term or long-term occlusion. The right action recognized is shown in Figure 4. Besides, the comparison of each method is also shown in Figure 5.

**Figure 6:**
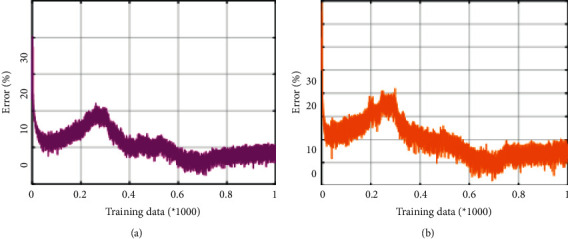
Error comparison. (a) Improved model and (b) original model.

This paper adopts the cascade matching strategy proposed by Wojke to deal with this association matching problem. The cascade matching strategy first records the number of unmatched frames (age) for each track. If a track does not find the corresponding detection target to match it within *k* frames, its age value is recorded as *k*, and one is in the current frame. When the frame finds a matching track, the age value is set to 0. Cascading matching firstly matches the trajectory with a smaller age value to the detection in the current frame, that is, the trajectories with an age from 0 to the preset threshold are matched with the detection of the current frame one by one, and the trajectories that have not been lost are preferentially matched. Longer trajectories are matched later. By processing in this way, the occluded target can be retrieved again, and the number of identity exchanges of the target that reappears after being occluded can be reduced. In addition, the main basis of matching is the above-mentioned four types of input information (Malanlan distance, intersection ratio, contextual features, and individual features). Among them, Mahalanobis distance and intersection ratio are only valid when age = 0. This is because if a trajectory loses a certain number of frames, the reliability of its trajectory prediction value is poor, so only the trajectory that has not been lost is used for Mahalanobis. For the lost track (age >0), the context feature is preferred to calculate the similarity matrix, but after more than 3 frames are lost, it is considered that the context feature of the player will also change due to the changes in the adjacent players, so it is used instead. The error comparison is shown in Figure 6.

## 4. Experiments

**Figure 7:**
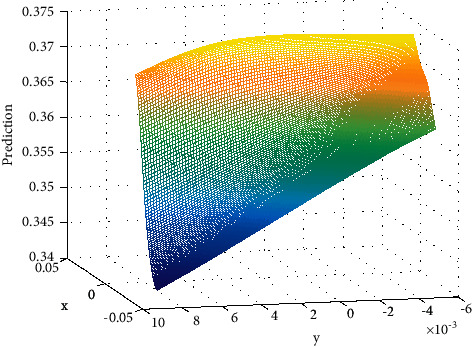
Prediction.

In order to verify the effectiveness of the multiplayer tracking algorithm based on the context graph model proposed in this chapter, this paper conducts quantitative and qualitative tests on the APIDIS basketball public data set and the data set collected by our research group. Since public data sets provide true values, quantitative analysis and comparison of related methods can be facilitated. The APIDS basketball game public data set is collected by 7 simultaneous cameras, including 5 ground cameras and 2 fisheye cameras, each set to a frame rate of 25 fps and a resolution of 800 ∗ 600. The data set scene includes a total of 10 players to be tracked. The difficulty of this data set is that the players in the same team look very similar, and the lighting conditions of the entire stadium are poor. In this experiment, two 1500-frame-long image frames captured by camera 1 and camera 6 are used as test data, and other image sequences are used as training data to train the player's context graph model. In addition to the basketball game data set, the research group also collected two indoor pedestrian data sets to verify the applicability of the multitarget tracking method proposed in this chapter to tracking problems in research fields such as intelligent monitoring and mobile robots. One set is a monitoring data set shot by a fixed camera, including a total of 4 people, but the occlusion phenomenon is serious, and the total test frame length is 500 frames; the other set is a data set shot by a camera mounted on a mobile robot, which has a total of 5 people; because the main goal of the mobile camera is to follow a specific person, only 1 person appears in the shot for a long time, and the rest of the people enter and exit the shot more frequently. The total test frame length of this data set is 1700 frames. The prediction is shown in Figure 7.

Since the selected data set provides two-dimensional real trajectories of players, quantitative comparison experiments are conducted on the data set. The general content of the experiment will be introduced as follows.

### 4.1. Quantitative Experimental Tests on Public Data Sets

This section mainly does the following three tasks on the public data set:

- Create a context graph for each player based on the real player trajectory on the selected data set and train the RBFNN model.
- Using three different player detection results, the multiplayer tracking based on the above RBF neural network model is realized respectively.
- Two detection-based multitarget tracking methods (DeepSort and PTSN) are replicated in the selected data set. DeepSort is the benchmark method of the improved method in this paper, and PTSN is a new three-way network based on human posture information, which is specially proposed for multiplayer tracking in volleyball video.

Table 1 shows the quantitative comparison results of various methods on the selected data set. Among them, the RBFNN is the method proposed in this paper, which actually adds the motion constraint based on Mahalanobis distance and intersection ratio on the basis of context feature matching, thus further improving the performance of multiplayer tracking compared with other methods.

**Figure 8:**
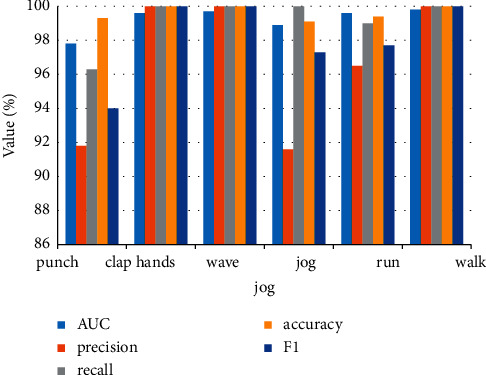
Different results.

**Figure 9:**
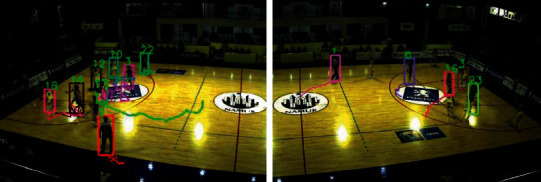
Different tracking effects.

It can be clearly seen from above that the introduction of context features significantly improves multiplayer tracking performance. For example, compared with the existing DeepSort and PTSN methods, the RBFNN method proposed in this chapter achieves better tracking performance. At the same time, the experimental results also show that these detection-based multitarget tracking methods listed in the table are sensitive to the detection results, and the better the detection effect is, the better the final tracking effect is. For the same detection result, compared with DeepSort method based on individual characteristics, the introduction of BaseGCN model improved its MOTA value from 56.0 to 59.6, and other evaluation indexes were also significantly improved. When the geometrical distance between adjacent players is further considered (DistGCN), the MOTA value is further increased to 60.4. Finally, multiplayer tracking accuracy (MOTA) reached a maximum of 63.0 when considering both contextual features and motion constraints (DistGCN + Motion). Under ideal conditions, if all the given detection frames are accurate (real detection values), the multiplayer tracking method RBFNN proposed in this chapter will get tracking effects very close to the real track (MOTA value is as high as 99.6, and the number of identity exchange is 0).  Figure 8 shows different results, and Figure 9 shows the different tracking effects corresponding to different detections. It can be clearly seen that the tracking effect is the worst, while the tracking effect based on real detection values is the best, which is consistent with the quantitative comparison results shown earlier.

## 5. Conclusion

This paper applies it to the analysis of the human body information recognition model in sports. The research results show that the human body information recognition model based on RBFNN proposed in this paper has a high recognition accuracy and can detect the movement state of people in sports in real time and accurately.

In sports, since the movement of the human body is composed of the complexity of the movement of the human body, which leads to the movement of the whole body, it is diverse and complicated, so the accurate tracking of the human movement is not easy to achieve. Therefore, it is necessary to study other more accurate feature extraction methods.

## Figures and Tables

**Table 1:** Performance comparison.

	MOTA↑	MOTP↑	FP↓	FN↓	IDs↓	FM↓
DeepSort	22.0	68.3	2315	3494	267	525
PTSN	24.7	68.5	2298	3316	250	522
BaseGCN	28.5	68.7	2164	3156	247	476
DistGCN	30.7	68.9	2108	3070	216	468
RBFNN (our)	39.8	69.0	1783	2764	146	431

## Data Availability

The data used to support the findings of this study are available from the corresponding author upon request.
